# Proteomic profiling and integrated analysis with transcriptomic data bring new insights in the stress responses of *Kluyveromyces marxianus* after an arrest during high-temperature ethanol fermentation

**DOI:** 10.1186/s13068-019-1390-2

**Published:** 2019-03-09

**Authors:** Pengsong Li, Xiaofen Fu, Ming Chen, Lei Zhang, Shizhong Li

**Affiliations:** 1 MOST-USDA Joint Research Center for Biofuels, Beijing Engineering Research Center for Biofuels, Institute of New Energy Technology, Tsinghua University, Beijing, 100084 China; 2 Agricultural Utilization Research Center, Nutrition and Health Research Institute, COFCO Corporation, No.4 Road, Future Science and Technology Park South, Beiqijia, Changping, Beijing, 102209 China

**Keywords:** *Kluyveromyces marxianus*, Proteome, Correlation analysis, High-temperature fermentation, Stress response

## Abstract

**Background:**

The thermotolerant yeast *Kluyveromyces marxianus* is a potential candidate for high-temperature fermentation. When *K. marxianus* was used for high-temperature ethanol fermentation, a fermentation arrest was observed during the late fermentation stage and the stress responses have been investigated based on the integration of RNA-Seq and metabolite data. In order to bring new insights into the cellular responses of *K. marxianus* after the fermentation arrest during high-temperature ethanol fermentation, quantitative proteomic profiling and integrated analysis with transcriptomic data were performed in this study.

**Results:**

Samples collected at 14, 16, 18, 20 and 22 h during high-temperature fermentation were subjected to isobaric tags for relative and absolute quantitation (iTRAQ)-based proteomic profiling and integrated analysis with transcriptomic data. The correlations between transcripts and proteins for the comparative group 16 h vs 14 h accounted for only 4.20% quantified proteins and 3.23% differentially expressed proteins (DEPs), respectively, much higher percentages of correlations (30.56%–59.11%) were found for other comparative groups (i.e., 18 h vs 14 h, 20 h vs 14 h, and 22 h vs 14 h). According to Spearman correlation tests between transcriptome and proteome (the absolute value of a correlation coefficient between 0.5 and 1 indicates a strong correlation), poor correlations were found for all quantified proteins (*R* = − 0.0355 to 0.0138), DEPs (*R* = − 0.0079 to 0.0233) and the DEPs with opposite expression trends to corresponding differentially expressed genes (DEGs) (*R* = − 0.0478 to 0.0636), whereas stronger correlations were observed in terms of the DEPs with the same expression trends as the correlated DEGs (*R* = 0.5593 to 0.7080). The results of multiple reaction monitoring (MRM) verification indicate that the iTRAQ results were reliable. After the fermentation arrest, a number of proteins involved in transcription, translation, oxidative phosphorylation and fatty acid metabolism were down-regulated, some molecular chaperones and proteasome proteins were up-regulated, the ATPase activity significantly decreased, and the total fatty acids gradually accumulated. In addition, the contents of palmitic acid, oleic acid, C16, C18, C22 and C24 fatty acids increased by 16.77%, 28.49%, 14.14%, 26.88%, 628.57% and 125.29%, respectively.

**Conclusions:**

This study confirmed some biochemical and enzymatic alterations provoked by the stress conditions in the specific case of *K. marxianus*: such as decreases in transcription, translation and oxidative phosphorylation, alterations in cellular fatty acid composition, and increases in the abundance of molecular chaperones and proteasome proteins. These findings provide potential targets for further metabolic engineering towards improvement of the stress tolerance in *K. marxianus*.

**Electronic supplementary material:**

The online version of this article (10.1186/s13068-019-1390-2) contains supplementary material, which is available to authorized users.

## Background

Bioethanol is becoming increasingly important, because it can reduce emissions of net CO_2_ and air pollutant [1–3]. Large-scale bioethanol production benefits greatly from high-temperature fermentation (≥ 40 °C), which can significantly reduce cooling costs and help prevent contamination [4, 5]. In addition, a high operating temperature also benefits the simultaneous saccharification and fermentation (SSF) process, because the optimal temperature for thermophilic enzymes catalyzing the saccharification of biomass is usually over 50 °C [6–8]. High temperatures can seriously destroy cytoskeletal integrity, inhibit cell division and growth, and affect metabolic activity [9–11]. Thus, the thermotolerant yeast *Kluyveromyces marxianus*, which can grow at elevated temperatures (> 40 °C) [12–14], becomes a potential candidate for high-temperature ethanol fermentation.

During high-temperature ethanol fermentation, yeast is faced with a variety of inhibitors such as ethanol [15, 16], acetic acid [17] and reactive oxygen species (ROS) [18]. The mechanisms behind the cellular responses of yeast to stress conditions are complex and have not been fully understood. In recent years, the cellular responses of *K. marxianus* to stress conditions have been investigated based on RNA sequencing (RNA-Seq)-based transcriptomic analysis [16, 19, 20].

In recent years, proteomic analysis has become an effective method to analyze the global protein profile in yeasts [21–26]. It has been demonstrated that the integrated analysis between proteomic and transcriptomic data can achieve a more complete understanding of gene expression regulation and its relationship to cellular function [27–32]. With the advancement in quantitative mass spectrometry (MS), shotgun proteomics can provide a relatively high-throughput assessment of changes in protein expression [33]. Shotgun proteomic methods address some of the limitations of conventional gel-based proteomic approaches, namely, low quantitative reproducibility and the inability to analyze highly basic/hydrophobic proteins [33, 34]. The isobaric tags for relative and absolute quantitation (iTRAQ) technique, which is one of the methods for shotgun proteomics, has been proven to be a reliable proteomic method for yeast studies [24, 25, 35, 36]. As we know, however, no study has investigated the protein profile of the yeast *K. marxianus* based on the iTRAQ technique so far.

In a recent study, we found that when *K. marxianus* was used for high-temperature ethanol fermentation, there was nearly no difference in glucose consumption and ethanol production between 30 and 45 °C during the early and middle stages of fermentation, but a fermentation arrest was observed during the late fermentation stage at 45 °C [20]. Then we investigated the stress responses of *K. marxianus* base on the integration of RNA-Seq and metabolite data. However, it is thought that mRNA levels only explain less than half of the variability in protein levels [22], because protein abundance is controlled not only by regulating mRNA abundance, but also by mechanisms that affect other steps of protein metabolism, such as protein translation and degradation [37, 38]. Therefore, protein abundance cannot be directly inferred from corresponding mRNA abundance [27–31]. In order to gain deeper insights into the cellular responses of *K. marxianus* to the stress condition during the late stage of high-temperature ethanol fermentation, we performed iTRAQ-based quantitative proteomic analysis and integrated analysis with transcriptomic data in this study, and some mechanisms of stress responses were confirmed at the protein level.

## Results

### Overall proteome of *K. marxianus* during high-temperature ethanol fermentation

Protein samples that were collected at 14, 16, 18, 20 and 22 h of high-temperature fermentation (45 °C) were subjected to iTRAQ-based proteomic analysis with three biological replicates. A total of 951,425 spectra were generated from the tested samples, and 25,022 peptides and 3331 proteins were identified with the cutoff Mascot Percolator *q* value ≤ 0.01 (Additional file 2: Table S1). Detailed information such as protein mass, percent coverage, the number of peptides matching individual proteins, and accession numbers assigned to each of the identified proteins is listed in Additional file 2: Table S2. Overlaps between replicates are shown by a Venn diagram in Additional file 1: Fig. S1. As shown in the Venn diagram, 2303 proteins (which account for 69.1% of all identified proteins) were identified in all the three replicates; and 2771 proteins (which account for 83.2% of all identified proteins) were identified in at least two replicates.

Functional annotation of all the identified proteins was conducted based on the annotation information from the Gene Ontology (GO) database [39, 40] and the Kyoto Encyclopedia of Genes and Genomes (KEGG) database [41, 42]. We obtained 2219 and 2561 annotations from the GO database and the KEGG database, respectively (Additional file 2: Tables S2, S3 and Additional file 1: Fig. S2). Such annotation information provided a good foundation as a reference for subsequent analysis.

### The proteome alterations after the fermentation arrest

Since the fermentation arrest occurred at the 16th h of fermentation, we first examined the differences in the protein profile between the samples collected before and after the fermentation arrest (i.e., 16 h vs 14 h; 18 h vs 14 h; 20 h vs 14 h; 22 h vs 14 h). Compared with the 14-h samples, the samples collected at 16, 18, 20 and 22 h were identified to have 31 (27 down-regulated and 4 up-regulated), 216 (195 down-regulated and 21 up-regulated), 362 (334 down-regulated and 28 up-regulated) and 428 (372 down-regulated and 56 up-regulated) differentially expressed proteins (DEPs), respectively (fold change > 1.2, *q*-value < 0.05; Additional file 1: Fig. S3 and Additional file 2: Tables S4–S7). The number of DEPs increased with time (Additional file 1: Fig. S4), indicating that the influence of accumulated inhibitors on cell metabolism gradually increased. To understand the relationships and discrepancy of samples more intuitively and comprehensively, we conducted cluster analysis using the union set of the DEPs identified above. The same type of genes were gathered in a cluster with similar biological functions. According to the result of cluster analysis (Additional file 1: Fig. S5), the protein expression patterns of 20-h and 22-h samples were the most similar to each other and these two samples had moderate similarity to the 18-h sample in protein expression. The similarity between the 16-h sample and the other three samples was the lowest, indicating that 16 h was a transition stage of the metabolic inhibition and the proteome was altered dramatically after 16 h. We used the coefficient of variation (*CV*), which is defined as the ratio of the standard deviation (*SD*) to the mean value, to evaluate the reproducibility of repetitive samples. As shown in Additional file 1: Fig. S6, proteins with *CV* values less than 20% accounted for around 95% in all the four comparisons, indicating that our experiments by iTRAQ had high precision in protein quantitation.

Then we conducted GO enrichment analyses based on the DEP data and all the significantly enriched GO terms are listed in Additional file 2: Tables S8–S11. Given the relatively high similarity in protein profile between the samples taken from 18, 20 and 22 h, we also identified the overlap of GO terms enriched in DEPs of these samples and found that a number of GO terms related to mitochondria organization, RNA polymerase and chaperone binding, etc., were enriched all together at 18, 20 and 22 h (Additional file 2: Table S12).

**Fig. 1:**
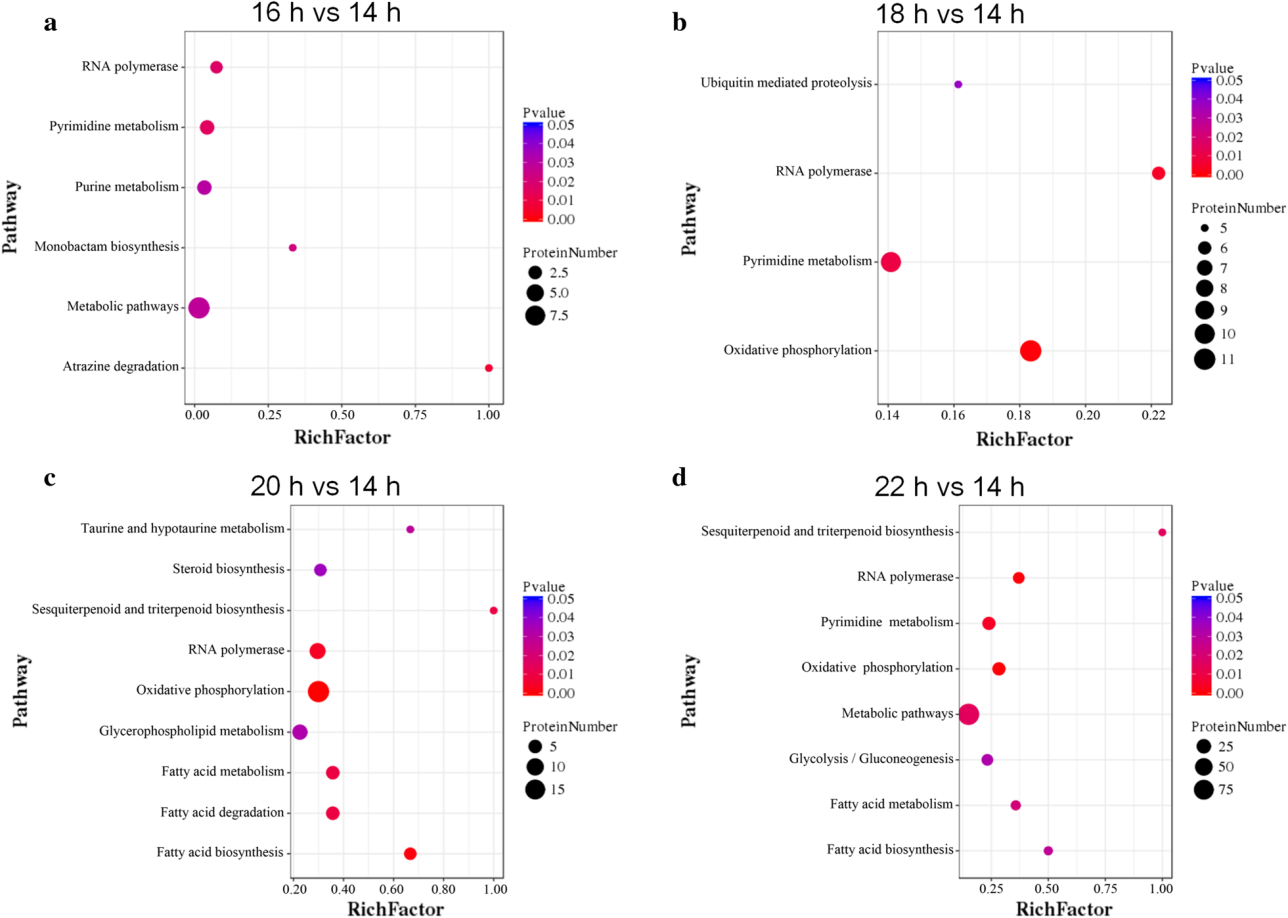
Statistics of KEGG pathway enrichment of DEPs in each pairwise. **a** 16 h vs 14 h; **b** 18 h vs 14 h; **c** 20 h vs 14 h; **d** 22 h vs 14 h. RichFactor is the ratio of DEP number annotated in this pathway term to all protein number annotated in this pathway term. Greater RichFator means greater effect of the inhibitors on the analyzed pathway

We also conducted pathway enrichment analyses of the DEPs based on the KEGG database in order to explore the changes of metabolic pathways after the fermentation arrest. At 16 h, some pathways related to RNA polymerase, pyrimidine metabolism, purine metabolism, monobactam biosynthesis and atrazine degradation were significantly enriched (Fig. 1a); at 18 h, some pathways involved in RNA polymerase, pyrimidine metabolism, ubiquitin-mediated proteolysis and oxidative phosphorylation were significantly enriched (Fig. 1b); at 20 and 22 h, pathways associated with RNA polymerase, fatty acid metabolism, steroid metabolism, oxidative phosphorylation, etc., were significantly enriched (Fig. 1c, d).

### Integrated analysis between proteome and transcriptome

**Fig. 2:**
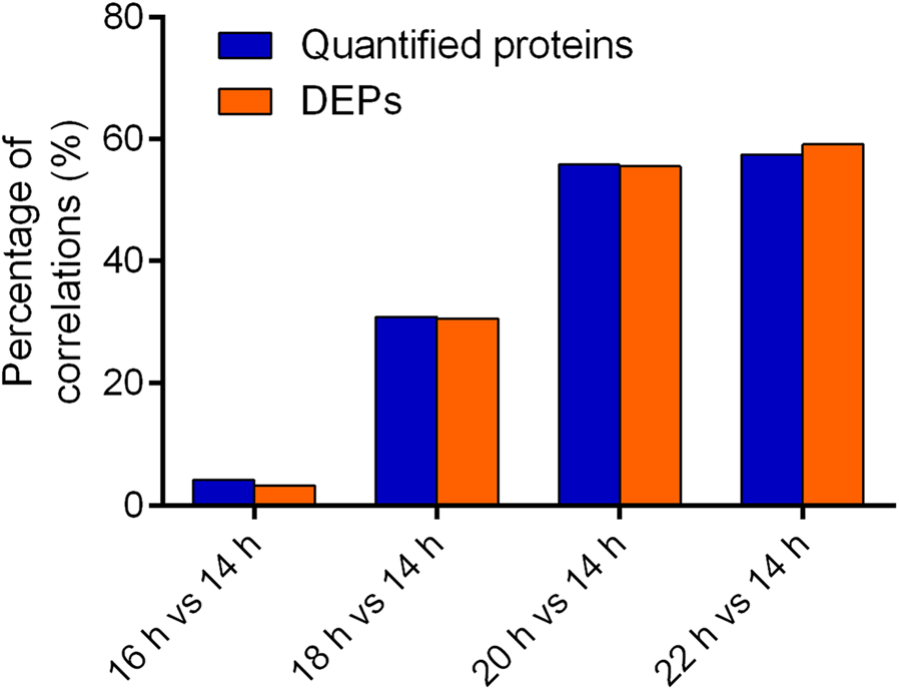
Percentage of correlations in quantified proteins and differentially expressed proteins (DEPs)

**Fig. 3:**
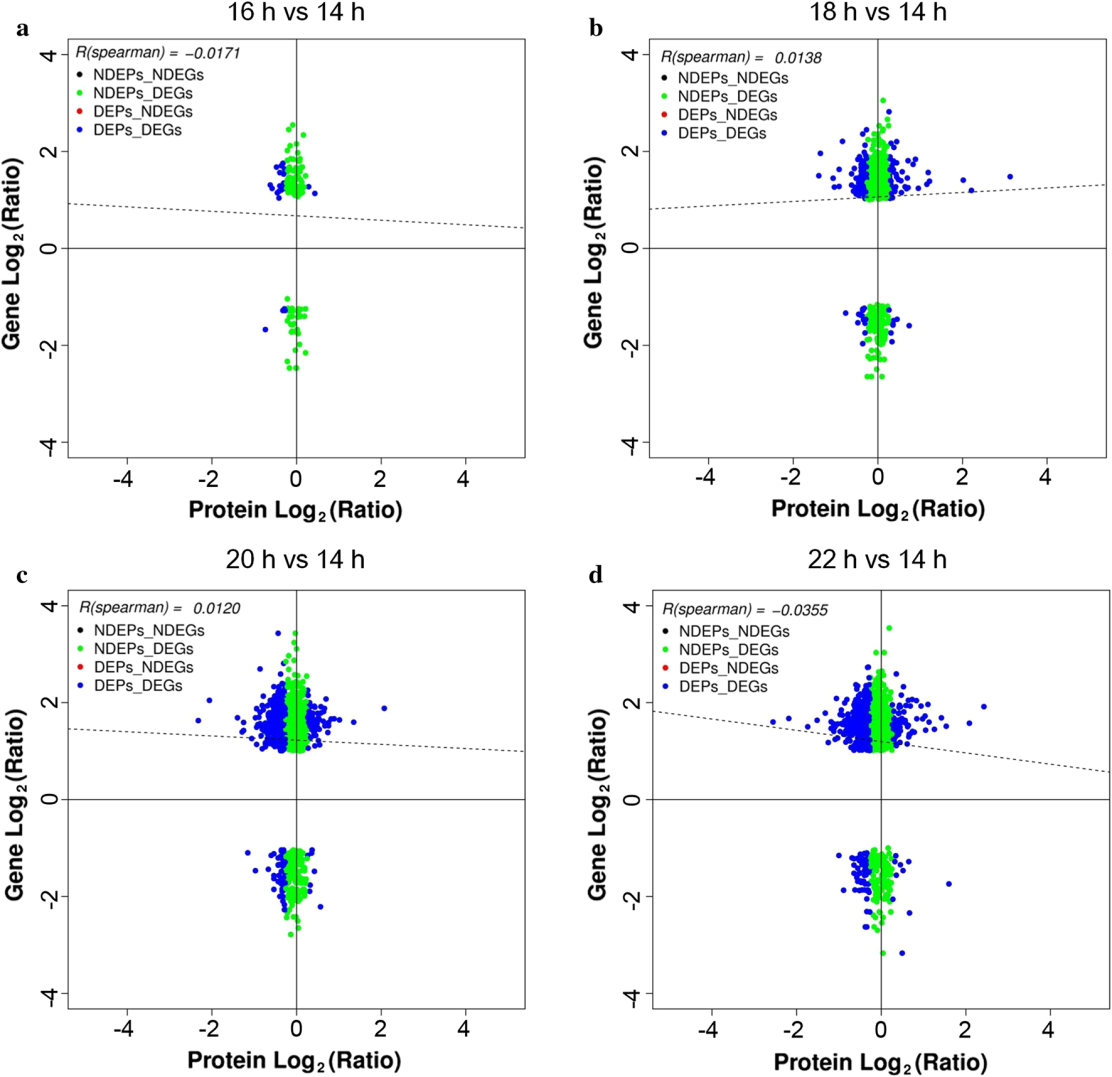
Comparison of expression ratios from transcriptomic (y-axis) and proteomic (x-axis) profiling based on quantitative proteins and the correlated genes in each pairwise. **a** 16 h vs 14 h; **b** 18 h vs 14 h; **c** 20 h vs 14 h; **d** 22 h vs 14 h. Significant expression changes were labeled in colors: red point, proteins only; green point, transcripts only; blue point, both; black, neither. The absolute value of an *R(Spearman)* between 0.5 and 1 indicates a strong correlation. The greater the absolute value of *R(Spearman)*, the stronger the correlation

To investigate whether the changes in protein abundance correlated with those in the corresponding mRNA level, we compared the expression changes in the transcriptome and proteome of different samples. Overlaps between DEPs and differentially expressed genes (DEGs) are shown by Venn diagrams in Additional file 1: Fig. S7. As shown in the Venn diagrams, there were considerable non-overlaps between DEPs and DEGs. The percentages of correlations in all quantified proteins and DEPs for all the comparative groups were shown in Fig. 2. For the comparative group 16 h vs 14 h, the percentages of correlations in quantified proteins and DEPs were as low as 3.23% and 4.20%, respectively (Fig. 2). For the comparative group 18 h vs 14 h, more than 30% of correlations in quantified proteins and DEPs were found, while more than 55% correlations in quantified proteins and DEPs were observed for both of the comparative groups of 20 h vs 14 h and 22 h vs 14 h (Fig. 2). According to Spearman correlation tests between transcriptome and proteome, poor correlations were found for all quantified proteins (*R* = − 0.0355 to 0.0138, Fig. 3), DEPs (*R* = − 0.0079 to 0.0233, Additional file 1: Fig. S8) and the DEPs with opposite expression trends to DEGs (*R* = − 0.0478 to 0.0636, Additional file 1: Fig. S9). However, stronger correlations were observed in terms of the DEPs with the same expression trends as the correlated DEGs except for 16 h vs 14 h (*R* = 0.5593 to 0.7080, Additional file 1: Fig. S10). Given that no correlation was found between the DEPs and corresponding DEGs with the same expression trends for 16 h vs 14 h, Spearman correlation test could not be conducted for this comparative group. According to the functional annotation of the DEPs with the same expression trends as DEGs based on the GO and KEGG database, the top functional categories, into which these proteins were categorized, were the GO terms “catalytic activity”, “binding”, “cell part”, “cell”, “metabolic process”, “cellular process”, etc. (Additional file 1: Figs. S11–S13) and the KEGG pathways “metabolic pathways”, “biosynthesis of secondary metabolites”, “biosynthesis of antibiotics”, etc. (Additional file 2: Tables S13–S15) for all the comparative groups 18 h vs 14 h, 20 h vs 14 h, and 22 h vs 14 h.

To explore the correlations between proteome and transcriptome from the perspective of protein function, we also conducted integrated analyses based on the results of DEP and DEG functional enrichment analyses. The significantly enriched (*p* < 0.05) GO terms and KEGG pathways for either proteome or transcriptome are listed in Additional file 2: Tables S16–S21. Based on these integrated analyses, we found that most of the GO terms and KEGG pathways significantly enriched in DEGs were not significantly enriched in DEPs except for the following exceptions: for 18 h vs 14 h, the GO terms “mitochondrial respiratory chain”, “identical protein binding” and “negative regulation of catalytic activity” were significantly enriched in both DEPs and DEGs, while the KEGG pathways “oxidative phosphorylation” and “ubiquitin mediated proteolysis” were significantly enriched only in DEPs; for 20 h vs 14 h, the KEGG pathway “steroid biosynthesis” and the GO terms “transmembrane transporter activity”, “oxidoreductase activity” and “cellular response to oxidative stress” were significantly enriched in both DEPs and DEGs, while the KEGG pathways “sesquiterpenoid and triterpenoid biosynthesis” and “taurine and hypotaurine metabolism” were significantly enriched only in DEPs; for 22 h vs 14 h, the GO term “oxidoreductase activity” was significantly enriched in both DEPs and DEGs, while the KEGG pathways “oxidative phosphorylation”, “pyrimidine metabolism”, “metabolic pathways” and “sesquiterpenoid and triterpenoid biosynthesis” were significantly enriched only in DEPs.

### Transcription and translation after the fermentation arrest

**Fig. 4:**
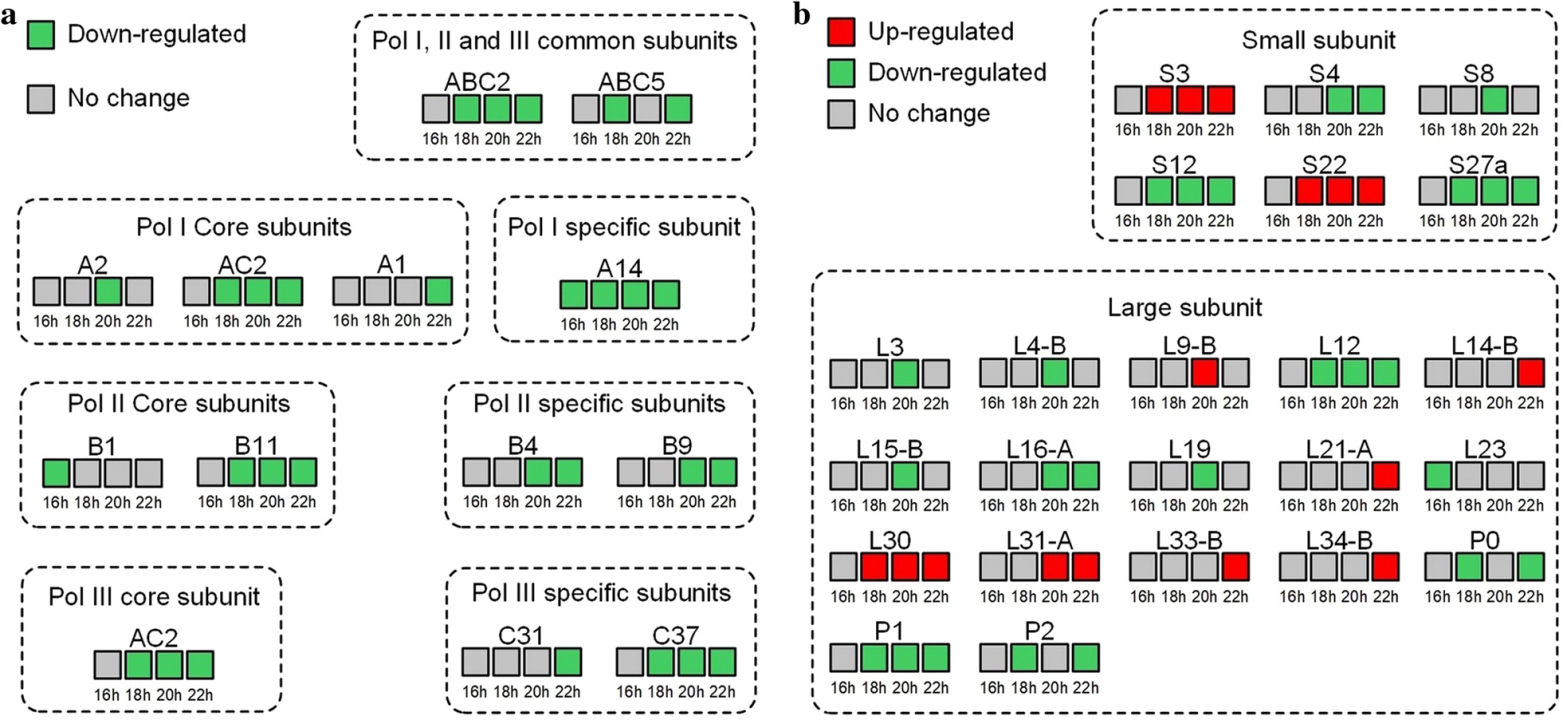
Differentially expressed proteins (DEPs) composing **a** RNA polymerase I, II and II and **b** ribosome

Changes in the abundance of some proteins involved in transcription and translation were found after the fermentation arrest. As shown in Fig. 4a, a majority of proteins that compose the RNA polymerase I, II and III (such as ABC2, ABC5, AC2, A14, B11, B4, B9 and C37) were down-regulated at no less than two time points after the fermentation arrest (Fig. 4a). Some ribosome proteins that compose the small ribosomal subunit (such as S4, S12 and S27a) and the large ribosomal subunit (such as L12, L6-A, P0, P1 and P2) were also down-regulated at no less than two time points after the fermentation arrest (Fig. 4b). On the contrary, we also observed increased abundance of some proteins that compose the small ribosomal subunit (such as S3 and S22) and the large ribosomal subunit (such as L30 and L31-A) at no less than two time points after the fermentation arrest.

### Oxidative phosphorylation after the fermentation arrest

**Fig. 5:**
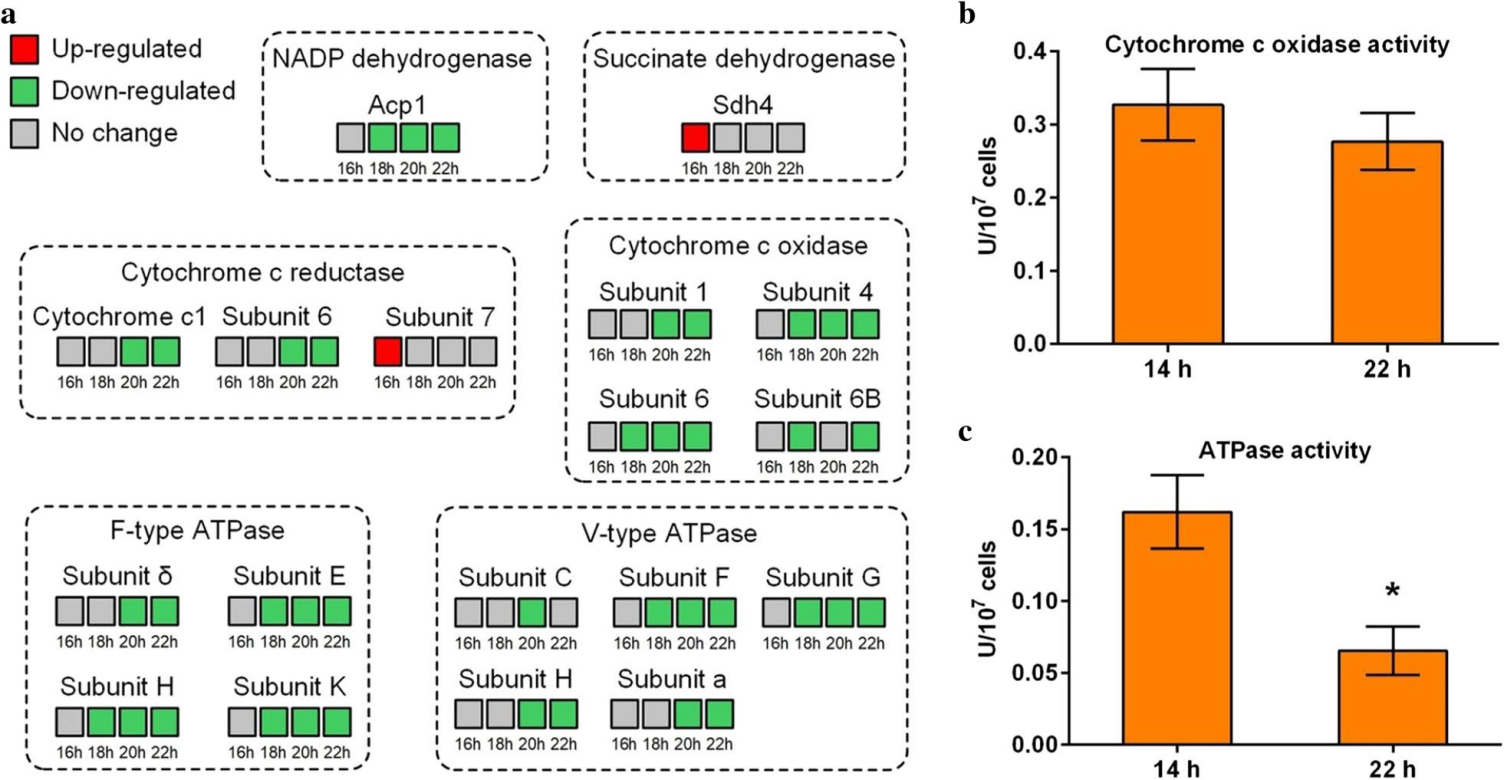
**a** Differentially expressed proteins (DEPs) related to oxidative phosphorylation; **b** cytochrome c oxidase activity at 14 and 22 h; **c** ATPase activity at 14 and 22 h. Values in **b** and **c** are means and standard deviations (n = 3). *Significantly different from the 14-h values (*p* < 0.05)

As shown in Fig. 5a, some proteins associated with oxidative phosphorylation were differentially expressed after the fermentation arrest. Among these proteins, many were down-regulated at no less than two time points after the fermentation arrest, including the NADP dehydrogenase-composing protein Acp1, the cytochrome c1 and subunit 6 of cytochrome c reductase, the subunits 1, 4, 6 and 6B of cytochrome c oxidase, the subunits δ, E, H and K of F-type ATPase and the subunits F, G H and a of V-type ATPase (Fig. 5a). All these results indicate repressed oxidative phosphorylation activity.

We also performed cytochrome c oxidase and ATPase activity assays to verify the iTRAQ results. There was no significant difference between the cytochrome c oxidase activity at 14 h and that at 22 h (Fig. 5b), whereas the ATPase activity significantly decreased from 14 to 22 h (Fig. 5c) (*p* < 0.05).

### Molecular chaperones and proteasome proteins

As shown in Table 1, some molecular chaperones and proteasome proteins, which are, respectively, involved in refolding and degradation of proteins, were found to be up-regulated after the fermentation arrest. The total number of up-regulated chaperones and proteasome proteins exhibited an increasing trend with time (Table 1), indicating that the increased synthesis of these proteins might be one of the cellular stress responses to the gradually accumulated fermentation inhibitors. Among these proteins, Gtt1, Atx1, Rpn6 and Pre7 were up-regulated at no less than two time points after the fermentation arrest.

**Table 1:** Molecular chaperones and proteasome proteins with increased abundance after the fermentation arrest. Rep1, Rep2 and Rep3 represent biological replicates

Protein accession	Protein name	Fold change (Rep1|Rep2|Rep3)	Description
*18* *h vs 14* *h*			
XP_022676480.1	Gtt1	1.212|1.059|1.358	Glutathione *S*-transferase 1
XP_022677923.1	Pre1	1.231|1.298|1.050	Proteasome component C11
XP_022674972.1	Rpn6	1.121|1.311|1.480	26S proteasome regulatory subunit Rpn6
*20* *h vs 14* *h*			
XP_022673658.1	Atx1	1.195|1.395|1.280	Metal homeostasis factor Atx1
XP_022675082.1	Hsp12	1.260|1.132|1.309	12 kDa heat shock protein
XP_022674972.1	Rpn6	1.234|1.200|1.291	26S proteasome regulatory subunit Rpn6
XP_022677708.1	Pre7	1.310|1.289|1.093	Proteasome component C5
*22* *h vs 14* *h*			
XP_022677580.1	Cpr6	1.283|1.265|1.331	Peptidyl-prolyl cis–trans isomerase D
XP_022674802.1	Sis1	1.029|1.208|1.206	Protein Sis1
XP_022676480.1	Gtt1	1.414|1.002|1.391	Glutathione *S*-transferase 1
XP_022673658.1	Atx1	1.153|1.494|1.426	Metal homeostasis factor Atx1
XP_022674972.1	Rpn6	1.265|1.210|1.469	26S proteasome regulatory subunit Rpn6
XP_022676879.1	Rpn7	1.142|1.274|1.243	26S proteasome regulatory subunit Rpn7
XP_022677708.1	Pre7	1.241|1.193|1.204	Proteasome component C5

### Changes of fatty acid composition after the fermentation arrest

Decreases in the abundance of some proteins related to fatty acid metabolism were found after the fermentation arrest. In particular, the long-chain-fatty-acid-CoA ligase 1 (Faa1), the acetyl-CoA carboxylase (Acc1) and the fatty acid synthase subunits beta (Fas1) were down-regulated at both 20 and 22 h. Then we measured the contents of various fatty acids in the samples collected at 14 and 22 h. As shown in Table 2, C16 and C18 fatty acids accounted for more than 98% of the total fatty acids at both 14 and 22 h. The total fatty acid content at 22 h was 20.44% higher than that at 14 h. Palmitic acid (C16:0), oleic acid (C18:1n9c) and palmitoleic acid (C16:1) were the top three dominant fatty acids at both 14 and 22 h. It is interesting that from 14 to 22 h, the contents of C16:0 and C18:1n9c increased by 16.77% and 28.49%, respectively (*p* < 0.05), while that of C16:1 did not change significantly. Similarly, the total content of C16 fatty acids significantly increased by 14.14% (*p* < 0.05), while that of C18 fatty acids significantly increased by 26.88% (*p* < 0.05). The contents of C20, C22 and C24 fatty acids were relatively low at both time points. From 14 h to 22 h, the contents of C22 and C24 fatty acids increased by 628.57% and 125.29%, respectively, while no significant change was observed in the total content of C20 fatty acids.

**Table 2:** Fatty acid profiles at 14 and 22 h during high-temperature (45 °C) fermentation

Fatty acids (mg/g DW)	14 h	22 h	Change from 14 to 22 h (%)
C16:0	4.080 ± 0.172	4.764 ± 0.122	16.77*
C16:1	1.426 ± 0.090	1.521 ± 0.125	6.64
C18:0	0.620 ± 0.062	0.775 ± 0.077	25.00*
C18:1n9c	3.361 ± 0.134	4.319 ± 0.200	28.49*
C18:2n6c	0.690 ± 0.020	0.848 ± 0.022	22.83*
C18:3n3	0.030 ± 0.011	0.023 ± 0.002	− 22.22
C20:0	0.024 ± 0.002	0.044 ± 0.002	79.45*
C20:1n9	0.004 ± 0.003	0.008 ± 0.000	100.00
C20:2	0.002 ± 0.003	0.006 ± 0.001	280.00
C20:4n6	0.026 ± 0.002	0.009 ± 0.002	− 64.10*
C22:0	0.002 ± 0.004	0.017 ± 0.000	628.57*
C24:0	0.029 ± 0.003	0.065 ± 0.001	125.29*
Total C16	5.506 ± 0.260	6.285 ± 0.236	14.14*
Total C18	4.701 ± 0.097	5.965 ± 0.268	26.88*
Total C20	0.056 ± 0.007	0.067 ± 0.003	20.24
Total C22	0.002 ± 0.004	0.017 ± 0.000	628.57*
Total C24	0.029 ± 0.003	0.065 ± 0.001	125.29*
Total SFA	4.755 ± 0.226	5.664 ± 0.102	19.12*
Total UFA	5.540 ± 0.142	6.735 ± 0.163	21.58*
Total mono-UFA	4.792 ± 0.154	5.848 ± 0.147	22.05*
Total	10.295 ± 0.279	12.399 ± 0.263	20.44*

DW, dry weight; SFA, saturated fatty acid; UFA, unsaturated fatty acid; mono-UFA, monounsaturated fatty acid

* Significantly changed (*p* < 0.05)

### Multiple reaction monitoring (MRM) verification of the iTRAQ data

To verify the quantitative data derived from iTRAQ, we conducted label-free quantification of some candidate DEPs for 22 h vs 14 h based on MRM. The comparison of the MRM and iTRAQ data are shown in Additional file 2: Table S22. These data were used for linear regression analysis. As shown in Additional file 1: Fig. S14, the log ratios of the quantitative data from MRM were correlated with those from iTRAQ (*R*^2^ = 0.8809), indicating that the iTRAQ results were reliable.

## Discussion

In this study, proteomic analysis revealed that some proteins related to gene transcription and translation were down-regulated in *K. marxianus* after the fermentation arrest, which is consistent with the results of transcriptomic analysis [20]. This phenomenon was likely associated with higher abundance of chaperones serving as protein foldases/structure protectors during the late fermentation stage (Table 1). Repressed gene transcription and translation were also observed in *K. marxianus* after the exposure to ethanol [16, 26] and multiple inhibitors [19], and in *S. cerevisiae* under stress conditions [43, 44]. It is also assumed that lowered protein synthesis may prevent continued gene expression under potentially error-prone conditions and allow the turnover of existing mRNAs and proteins while gene expression is reprogrammed to deal with the stress [44]. As for the situation in this study, the intracellular ROS were significantly accumulated after the fermentation arrest [20], which might result in DNA damages. Therefore, we think the repression of transcription and translation could be a self-protective mechanism of the yeast to cope with the stress condition during the late fermentation stage in this study.

Decreases in the abundance of some proteins involved in oxidative phosphorylation were also observed after the fermentation arrest, which was verified by ATPase activity assays. This finding coincides with both the repressed respiration activity in our previous study [20] and the fermentation arrest in this study. In addition, down-regulation of genes related to oxidative phosphorylation was also observed in *S. cerevisiae* cultured from fully aerobic or oxygen-limited conditions to anaerobic conditions [45, 46]. Thus, the down-regulation of proteins related to oxidative phosphorylation is thought to be a response to the shift from respiratory or respirofermentative to fully fermentative metabolism in this study.

We also found that some molecular chaperones and proteasome proteins, which are involved in the protein quality control (PQC) system [47], were up-regulated after the fermentation arrest. The interplays of these proteins involved in the PQC system are responsible for the folding of proteins, refolding of misfolded proteins and degradation of misfolded and damaged proteins [47]. The PQC system is thought to be able to protect protein functions under stress conditions (e.g., in the presence of elevated ROS level) [48], because it keeps the balance between the folding and degradation of proteins. It is thought that the PQC system maintains the balance of the cellular protein pool through two main pathways: (i) molecular chaperones, which prevent aggregation and ensure the folding of proteins to their native state, and (ii) the cellular degradation machinery, which targets proteins for proteolysis [49]. As shown in Table 1, the molecular chaperones Gtt1 and Atx1 were up-regulated at no less than two time points after the fermentation arrest. The glutathione *S*-transferase 1 (Gtt1), which is one of the glutathione *S*-transferases in yeast [50], can catalyze the reduction of hydroperoxides [51], while Atx1 is a small metal homeostasis factor which can protect cells against reactive oxygen toxicity [52]. Thus, we think that the up-regulation of these molecular chaperones can prevent protein aggregation and ensure that proteins fold to their native state against the oxidative stress after the fermentation arrest in this study. In addition, the 26S proteasome regulatory subunit Rpn6 and the proteasome component C5 were also up-regulated at no less than two time points after the fermentation arrest (Table 1), which might help degrade the misfolded and damaged proteins under the stress condition. Increases in abundance of proteins involved in the PQC system were also observed in *K. marxianus* under various stress conditions [16, 26, 53]. Thus, we think that the up-regulation of the molecular chaperones and proteasome proteins is one of the mechanisms of *K. marxianus* to deal with the stress condition during the late fermentation stage in this study.

Alterations in cellular fatty acid composition were also observed after the fermentation arrest in this study. You et al. [54] have demonstrated that oleic acid can overcome the toxic effects of ethanol in cells of *S. cerevisiae*, because the incorporation of oleic acid into lipid membranes is thought to be able to decrease membrane fluidity and can thereby counteract the fluidizing effects of ethanol [54]. In a previous study, the increase in the proportion of monounsaturated fatty acids (mono-UFAs) and the concomitant decrease in saturated fatty acids were observed upon exposure of *S. cerevisiae* to ethanol stress, whereas non-*Saccharomyces* yeasts, such as *Issatchenkia occidentalis* and *Issatchenkia orientalis*, exhibited decrease in mono-UFAs when exposed to ethanol [55]. In contrast, Diniz et al. [16] found that although the expression of some gene-encoding enzymes related to unsaturated fatty acids (UFAs) decreased upon ethanol exposure, the UFA content in *K. marxianus* did not change under this stress. Lower percentage of UFAs in total fatty acids of *K. marxianus* was also observed at 45 °C than at 30 °C (53.82% vs 59.55%), indicating heat-repressed UFA biosynthesis [20]. In the present study, we found that from 14 h to 22 h, the cellular oleic acid content and total mono-UFA content increased by 28.49% and 22.05%, respectively. In addition, an increase in the total fatty acid content was also observed after the fermentation arrest in this study, which is also thought to be responsible for ethanol tolerance [56]. Given that the ethanol concentration was up to 52 g/L during the late stage of fermentation in this study, the alterations in fatty acid content might be a compensatory mechanism of *K. marxianus* in response to ethanol stress.

Recently, Alvim et al. [26] investigated the ethanol stress responses of *K. marxianus* CCT 7735 by proteomic and metabolomic analyses. Interestingly, although both the conditions (aerobic growth vs high-temperature fermentation) and the inhibitors (ethanol added exogenously vs multiple inhibitors accumulated during high-temperature fermentation) were different between their study and the present study, some responses such as decreased abundances of proteins related to translation and increased abundances of chaperones were observed in both of the studies, indicating that these proteomic changes are general responses of *K. marxianus* to various stress conditions.

In this study, the proteome at 16 h was the most similar to that at 14 h among those after the fermentation arrest. Thus, fewest DEPs were identified for the comparative group 16 h vs 14 h (Additional file 1: Fig. S4). Given that the 16th h was a transition phase from ethanol fermentation to the fermentation arrest, complex regulations from transcripts to proteins might exist at this time point. This was confirmed by the enriched GO terms in the DEGs, such as “RNA binding” and “protein metabolic process”. Therefore, low percentages of correlations in both quantitative proteins (4.20%) and DEPs (3.23%) were observed for 16 h vs 14 h (Fig. 2). More research is needed to explore these complex regulations. Although much higher percentages of correlations (30.56% to 59.11%) were found for other comparative groups (Fig. 2), poor correlations between transcriptome and proteome were found by Spearman correlation tests for both quantified proteins (*R* = − 0.0355 to 0.0138) and DEPs (*R* = − 0.0079 to 0.0233). These findings are consistent with the results of previous studies that found poor correlations between proteomes and transcriptomes in other species of yeasts such as *S. cerevisiae* [27, 28] and *Schizosaccharomyces pombe* [57]. Given that protein synthesis requires multiple steps including transcription, translation, and post-translation modification, the correlation between proteome and transcriptome is usually low (27–40%) [58] and false positives may be found by single-omic analysis. Therefore, multi-omic data can be mutually verified, reducing the false positives found by single-omic analysis. The integrated analysis of multi-omic data can also make up for the data problems resulting from data missing and noise in single-omic data analysis. With the fold-change cutoff of 1.2 (*q*-value < 0.05) for DEPs and 2 (*q*-value < 0.05) for DEGs, less DEPs were identified than DEGs, and therefore the proteomic analysis in this study provided more precise and concise information than transcriptomic analysis did. Some of the RNA-Seq results were verified through integrated analysis of the proteomic and transcriptomic data. For instance, the KEGG pathway “steroid biosynthesis” was significantly enriched in DEPs after the fermentation arrest in this study, which coincided with the results of RNA-Seq and ergosterol measurements in our previous study [20]. These results corroborate each other and suggest that enhancing ergosterol biosynthesis is one of the stress responses of *K. marxianus* after the arrest during high-temperature ethanol fermentation.

## Conclusions

We herein employed iTRAQ-based quantitative proteomic analysis and integrated analysis with transcriptomic data to study the stress responses of *K. marxianus* during the late stage of high-temperature ethanol fermentation. This study confirmed some biochemical and enzymatic alterations provoked by the stress conditions in the specific case of *K. marxianus*: such as decreases in transcription, translation and oxidative phosphorylation, alterations in cellular fatty acid composition, and increases in the abundance of molecular chaperones and proteasome proteins. These findings provide potential targets for further metabolic engineering towards improvement of the stress tolerance in *K. marxianus*.

## Methods

### Sample collection

Cell samples of *K. marxianus* DMKU3-1042 (purchased from NITE Biological Resource Center with the deposit number of NBRC 104275), which were collected from our previous study [20] and stored at − 80 °C, were used in this study. These samples were collected from batch ethanol fermentation experiments that were conducted at 45 °C in 100-mL serum bottles containing 30 mL fermentation medium (20 g/L yeast extract, 20 g/L peptone, 180 g/L glucose, 0.6 g/L (NH_4_)_2_SO_4_ and 0.15 g/L KH_2_PO_4_). The samples collected at 14, 16, 18, 20 and 22 h (5 mL cell suspension with OD_600_ ~ 17 for each sample) were used for protein extraction and iTRAQ-based quantitative proteomic analysis. In this study, three biological replicates were used and there were 15 samples in total. The samples collected before the fermentation arrest (14 h) were set as the control group, and the samples collected after the fermentation arrest (16, 18, 20 and 22 h) were set as the treatment group to study the proteome changes resulting from the stress condition during the late fermentation stage.

### iTRAQ-based protein identification and quantitation

The iTRAQ technology was used to investigate the proteome changes in this study. Protein extraction, digestion, peptide labeling, peptide fractionation, high performance liquid chromatography (HPLC) and mass spectrometer (MS) detection were conducted at Beijing Genomics Institute (BGI, Shenzhen, China). Protein samples were extracted with lysis buffer (8 M urea, 40 mM Tris–HCl, pH 8.5) containing 1 mM phenylmethanesulfonyl fluoride (PMSF) and 2 mM ethylenediaminetetraacetic acid (EDTA). With placing on ice for 5 min, 10 mM dithiothreitol (DTT, final concentration) was added to the samples. The suspension was sonicated at 200 W for 1 min, centrifuged at 4 °C, 25,000*g* for 20 min, and then incubated at 56 °C for 1 h. Subsequently, after cooling to room temperature, the protein samples were incubated with 55 mM iodoacetamide (IAM, final concentration) for 45 min in the dark room for alkylation.

The protein solution (100 μg) with 8 M urea was diluted 4 times with 100 mM triethylammonium bicarbonate (TEAB). Trypsin Gold (Promega, Madison, WI, USA) was used to digest the proteins with the ratio of protein: trypsin = 40:1 at 37 °C overnight. After trypsin digestion, peptides were desalted with a Strata X C18 column (Phenomenex, Torrance, CA, USA) and vacuum-dried according to the manufacturer’s protocol. Then the peptides were dissolved in 30 ul 0.5 M TEAB with vortexing and peptide labeling was performed with the iTRAQ Reagents—8plex Kit (Applied Biosystems, Foster City, CA, USA) according to the manufacturer’s protocol. The labeled peptides with different reagents were combined and desalted with a Strata X C18 column (Phenomenex, Torrance, CA, USA) and vacuum-dried according to the manufacturer’s protocol.

The labeled peptides were separated on a Shimadzu LC-20AB HPLC Pump system coupled with a high pH RP column, reconstituted with buffer A [5% acetonitrile (ACN), 95% H_2_O, adjust pH to 9.8 with ammonia] to 2 mL, loaded onto a column containing 5-μm particles (Phenomenex), and then separated at a flow rate of 1 mL/min with a gradient of 5% buffer B (5% H_2_O, 95% ACN, adjust pH to 9.8 with ammonia) for 10 min, 5–35% buffer B for 40 min, 35–95% buffer B for 1 min. The system was then maintained in 95% buffer B for 3 min and decreased to 5% buffer B within 1 min, and equilibrated with 5% buffer B for 10 min. Elution was monitored by measuring the absorbance at 214 nm, and fractions were collected every 1 min. The eluted peptides were pooled as 20 fractions and vacuum-dried.

For HPLC analysis, each fraction was resuspended in buffer C (2% ACN and 0.1% formic acid in water) and centrifuged at 20,000*g* for 10 min. The supernatant was loaded onto a C18 trap column 5 μL/min for 8 min using a LC-20AD nano-HPLC instrument (Shimadzu, Kyoto, Japan) by the autosampler. Then the peptides were eluted from the trap column and separated by an analytical C18 column (inner diameter 75 μm) packed in-house. The gradient was run at 300 nL/min starting from 8 to 35% buffer D (2% H_2_O and 0.1% formic acid in ACN) in 35 min, increased to 60% buffer D in 5 min, then maintained at 80% buffer D for 5 min, and finally returned to 5% buffer D in 0.1 min and equilibrated for 10 min.

The peptides separated from nano-HPLC were subjected into the tandem mass spectrometry Q Exactive (Thermo Fisher Scientific, San Jose, CA, USA) for DDA (data-dependent acquisition) detection by nano-electrospray ionization. The parameters for MS analysis were as follows: electrospray voltage: 1.6 kV; precursor scan range: 350–1600 m/z at a resolution of 70,000 in Orbitrap; MS/MS fragment scan range: > 100 m/z at a resolution of 17,500 in HCD mode; normalized collision energy setting: 27%; dynamic exclusion time: 15 s; automatic gain control (AGC) for full MS target and MS2 target: 3e6 and 1e5, respectively; the number of MS/MS scans following one MS scan: 20 most abundant precursor ions above a threshold ion count of 10,000.

The raw MS/MS data were converted into MGF format with the ProteoWizard tool msConvert [59], and the exported MGF files were searched with Mascot (v2.3.02) [60] against the protein database of *K. marxianus* (https://www.ncbi.nlm.nih.gov/genome/?term=Kluyveromyces+marxianus). Quantitative analysis of the peptides labeled with isobaric tags was performed with IQuant software [61]. Proteins with fold change greater than 1.2 and *q*-value less than 0.05 were determined as differentially expressed proteins (DEPs). Since three biological replicates were used in this study, final DEPs were defined in at least two replicates.

### MRM verification

MRM was employed to verify the iTRAQ results. For the MRM verification, 20 µg of the protein solution from each sample was diluted 4 times with 25 mM NH_4_CO_3_, digested with 1 µL of trypsin (0.5 µg/µL) and incubated overnight at 37 °C. The digestion was stopped by adding TFA to a concentration of 0.1%. Then the MRM verification experiments were conducted according to the method reported by Zi et al. [62]. The raw data of the MRM were processed using MultiQuant 2.0.2 (AB SCIEX). After manual inspection and retention alignment, peaks with an S/N > 10 were qualified for quantification. The comparison and scatter plot of the MRM and iTRAQ data were completed using Excel (Microsoft Office 2016, USA).

### Bioinformatic analysis

Protein annotation was performed based on the BLAST comparisons against GO [39, 40] and KEGG [41, 42] databases, using Blast2GO [63] and KAAS [64], respectively. To identify significantly enriched GO terms and KEGG pathways, enrichment analyses were performed based on the hyper-geometric test. The statistical significance was calculated by

1 $$p = 1 - \mathop \sum \limits_{i = 0}^{m - 1} \frac{{\left( {\begin{array}{*{20}c} M \\ i \\ \end{array} } \right)\left( {\begin{array}{*{20}c} {N - M} \\ {n - i} \\ \end{array} } \right)}}{{\left( {\begin{array}{*{20}c} N \\ n \\ \end{array} } \right)}},$$

where *N* is the number of all identified proteins which matched to GO terms (or KEGG pathways); *n* is the number of DEPs; *M* is the number of proteins which matched a certain GO term (or KEGG pathway); *m* is the number of DEPs which matched a certain GO term (or KEGG pathway). If the *p* value is less than 0.05, the analyzed GO term (or KEGG pathway) is considered to be significantly enriched in the DEPs.

To compare the concordance between proteome and transcriptome after the fermentation arrest, we performed correlation analysis based on the iTRAQ results in this study and the RNA-Seq results from the aspects of expression and functional enrichment. Spearman correlation coefficient *R(Spearman)* was calculated using the cor.test function in the statistics package R. The absolute value of an *R(Spearman)* between 0.5 and 1 indicates a strong correlation. The greater the absolute value of correlation coefficient, the stronger the correlation.

### Fatty acid measurement

For fatty acid measurement, yeast cells were collected by centrifugation (3000*g* for 5 min at 4 °C) and washed twice with distilled water. These samples were then frozen at − 80 °C and freeze-dried. Cell lysis, extraction of total lipids, and conversion to fatty acid methyl esters (FAMEs) were based on the protocol of a previous study [65]. Then the FAMEs were quantified with the gas chromatograph-flame ionization detector (GC-FID) method described by Li et al. [66].

### Cytochrome c oxidase and ATPase activity assays

The activities of cytochrome c oxidase and ATPase were determined by the mitochondrial respiratory chain complex IV activity assay kit (Solarbio, Beijing, China) and the mitochondrial respiratory chain complex V activity assay kit (Solarbio, Beijing, China) following the manufacturer’s instruction, respectively. One unit (U) of cytochrome c oxidase activity was defined as 1 nmol of reduced cytochrome c degraded by cytochrome c oxidase per minute, while one U of ATPase activity was defined as 1 nmol of inorganic phosphorus generated in the reaction system per minute. The activities of cytochrome c oxidase and ATPase were expressed as U/10^7^ cells.

## Additional files

**Additional file 1.** Additional figures in this study.

**Additional file 2.** Additional tables in this study.

## Abbreviations

ACN: acetonitrile
CV: coefficient of variation
DEG: differentially expressed gene
DEP: differentially expressed protein
EDTA: ethylenediaminetetraacetic acid
FAME: fatty acid methyl ester
GO: gene ontology
HPLC: high-performance liquid chromatography
iTRAQ: isobaric tags for relative and absolute quantitation
KEGG: Kyoto Encyclopedia of Gene and Genomics
mono-UFA: monounsaturated fatty acid
MRM: multiple reaction monitoring
MS: mass spectrometry
PMSF: phenylmethanesulfonyl fluoride
RNA-Seq: ribonucleic acid (RNA) sequencing
SD: standard deviation
SFA: saturated fatty acid
TEAB: triethylammonium bicarbonate
UFA: unsaturated fatty acid
