# Supplementary material for: Proteomic profiling and integrated analysis with transcriptomic data bring new insights in the stress responses of Kluyveromyces marxianus after an arrest during high-temperature ethanol fermentation
Source: Biotechnol Biofuels. 2019 Mar 9;12:49. doi: 10.1186/s13068-019-1390-2 (PMC6408782; doi:10.1186/s13068-019-1390-2)
Supplement: Supplementary file 1 — Additional file 1. Additional figures in this study. [file 13068_2019_1390_MOESM1_ESM.docx]

**Supplemental figures for**

Proteomic profiling and integrated analysis with transcriptomic data bring new insights in the stress responses of *Kluyveromyces marxianus* after an arrest during high-temperature ethanol fermentation

*Pengsong Li^a^, Xiaofen Fu^a^, Ming Chen^a^, Lei Zhang^a,b^, Shizhong Li^a,*^*

^a^ MOST-USDA Joint Research Center for Biofuels, Beijing Engineering Research Center for Biofuels, Institute of New Energy Technology, Tsinghua University, Beijing 100084, China

^b^ Agricultural Utilization Research Center, Nutrition and Health Research Institute, COFCO Corporation, No.4 Road, Future Science and Technology Park South, Beiqijia, Changping, Beijing 102209, China

^*^Corresponding author at Institute of New Energy Technology, Tsinghua University, Beijing 100084, China. Tel.: +86-10-62772123. Fax: +86-10-80194050. Email: [szli@tsinghua.edu.cn](mailto:szli@tsinghua.edu.cn)

**There are 14 supplementary figures**


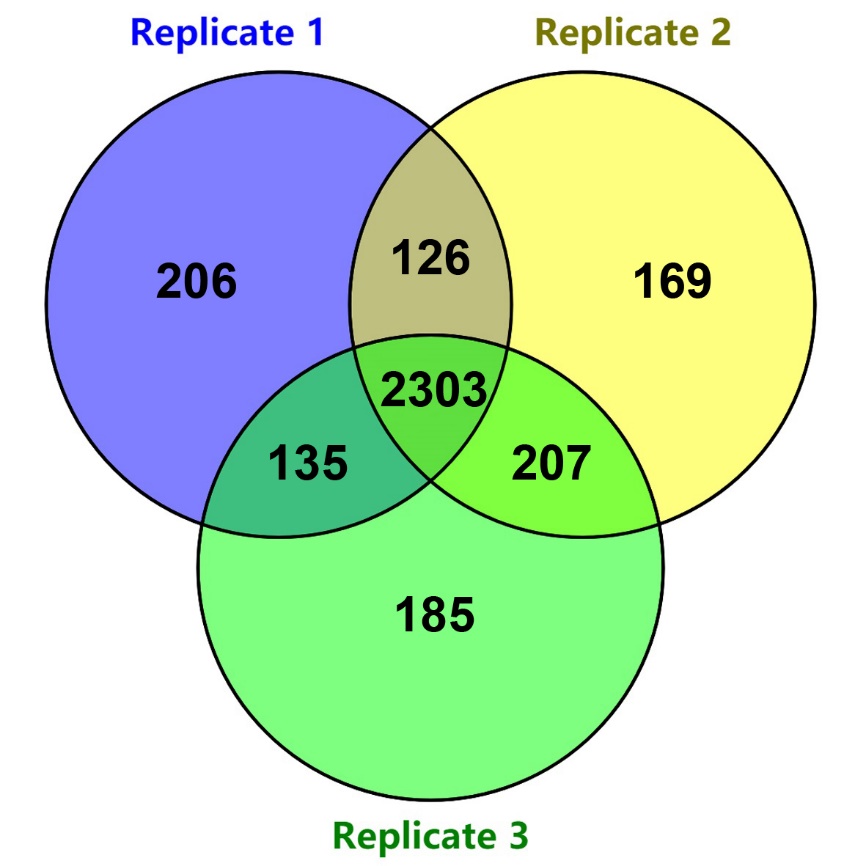


**Fig. S1** Venn diagram showing overlaps of identified proteins between replicates


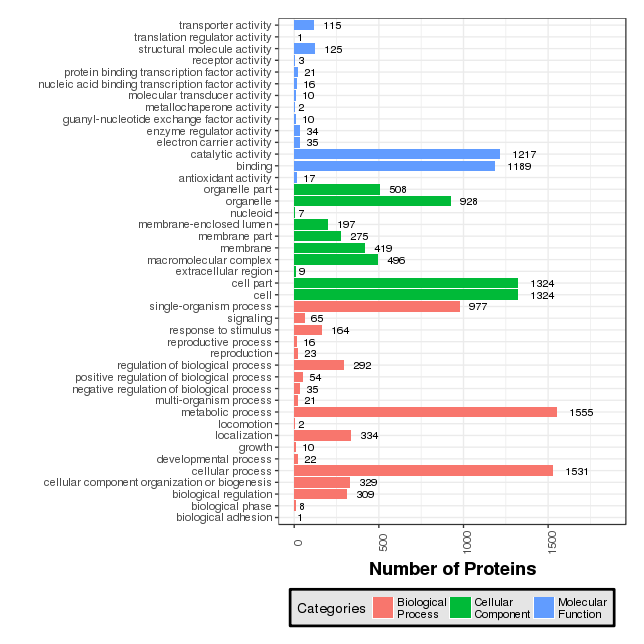


**Fig. S2** The distribution of GO terms according to GO annotation of all quantified proteins


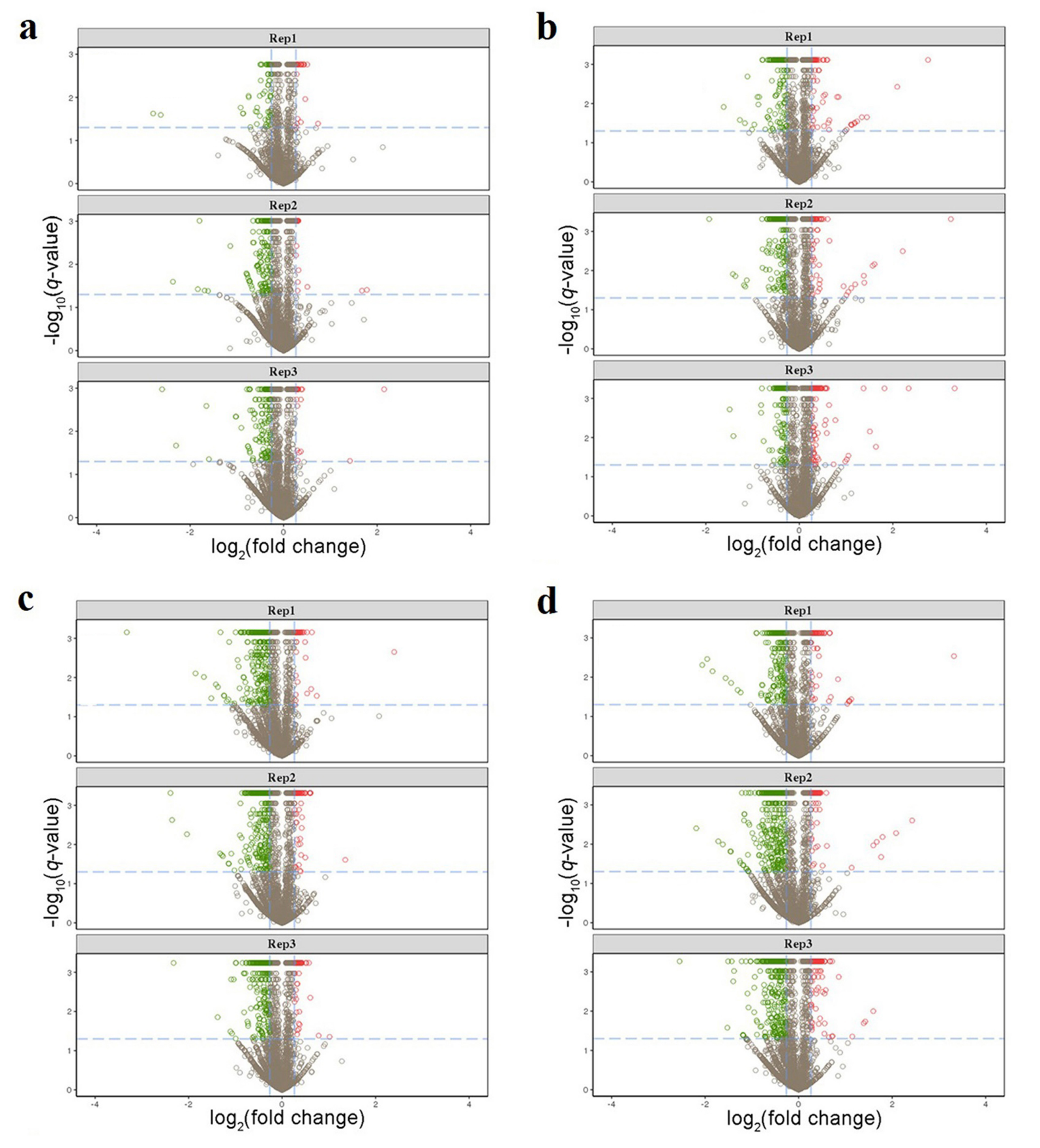


**Fig. S3** Volcano plots of differentially expressed proteins (DEPs) in each pairwise. **a** 16h vs 14h; **b** 18h vs 14h; **c** 20h vs 14h; **d** 22h vs 14h. Fold change > 1.2 and *q*-value < 0.05 were set as the significant threshold for differentially expression. Rep1, Rep2 and Rep3 represent the biological replicates in this study. Red circle, significant up-regulated proteins; green circle, significant down-regulated proteins; grey circle, proteins with no significant difference in abundance.


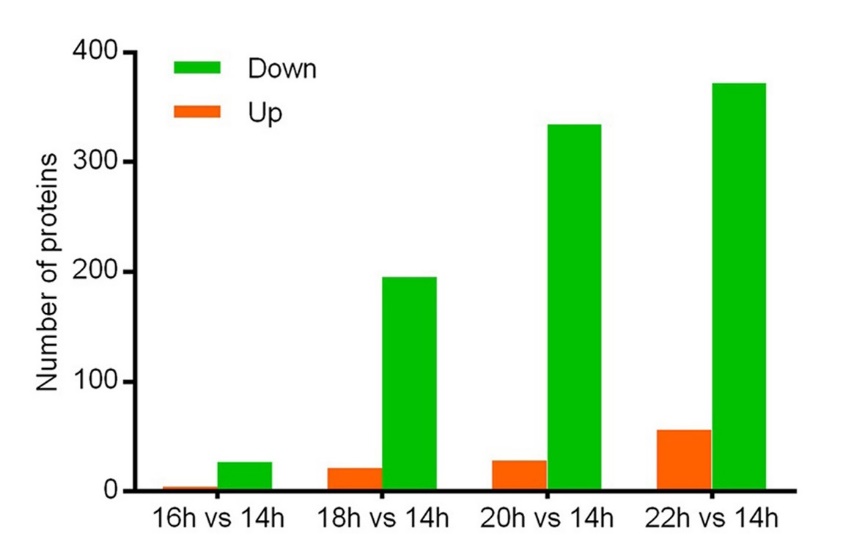


**Fig. S4** The numbers of DEPs for various comparative groups


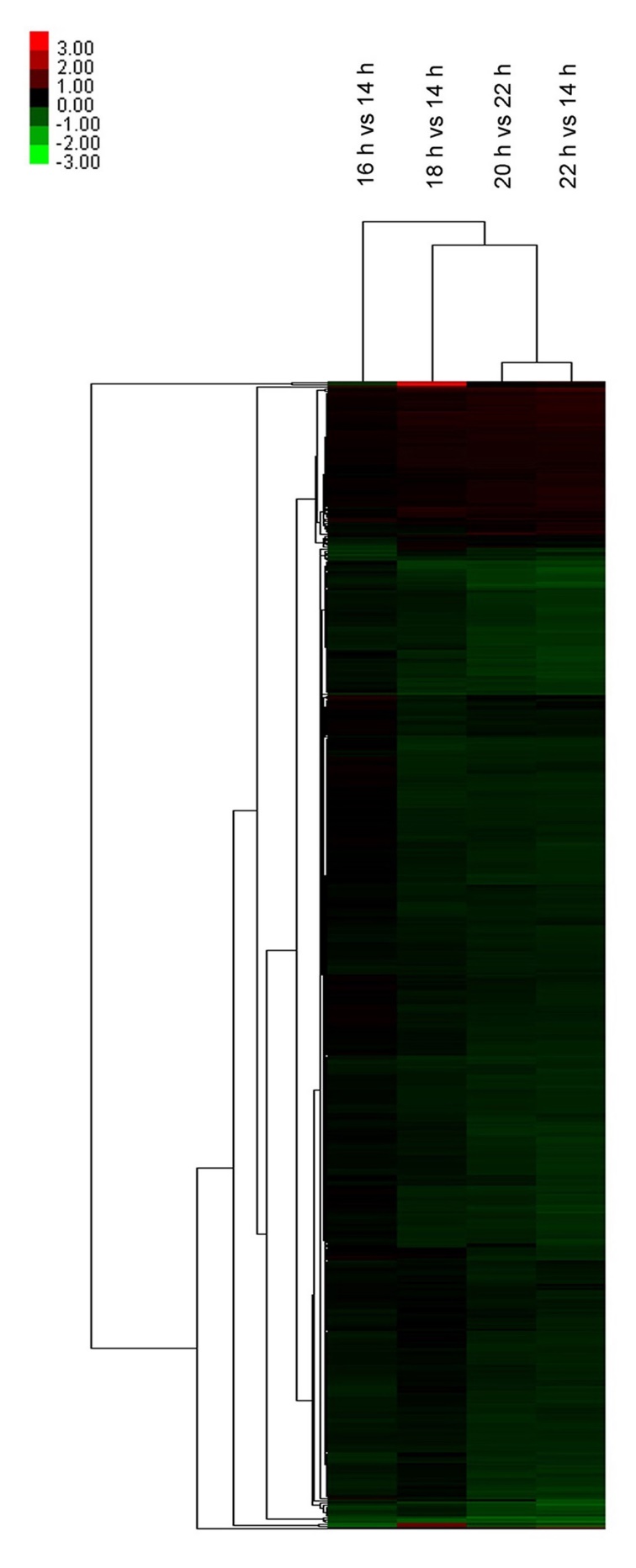


**Fig. S5** Cluster heat map showing the similarity between the proteome profiles at different time points after the fermentation arrest. The union set of the DEPs for all the comparative groups was used for cluster analysis. Cluster analysis was conducted based on Euclidean distance and hierarchical algorithm. The heatmap.2 function in R package gplots was used to generate the heat map


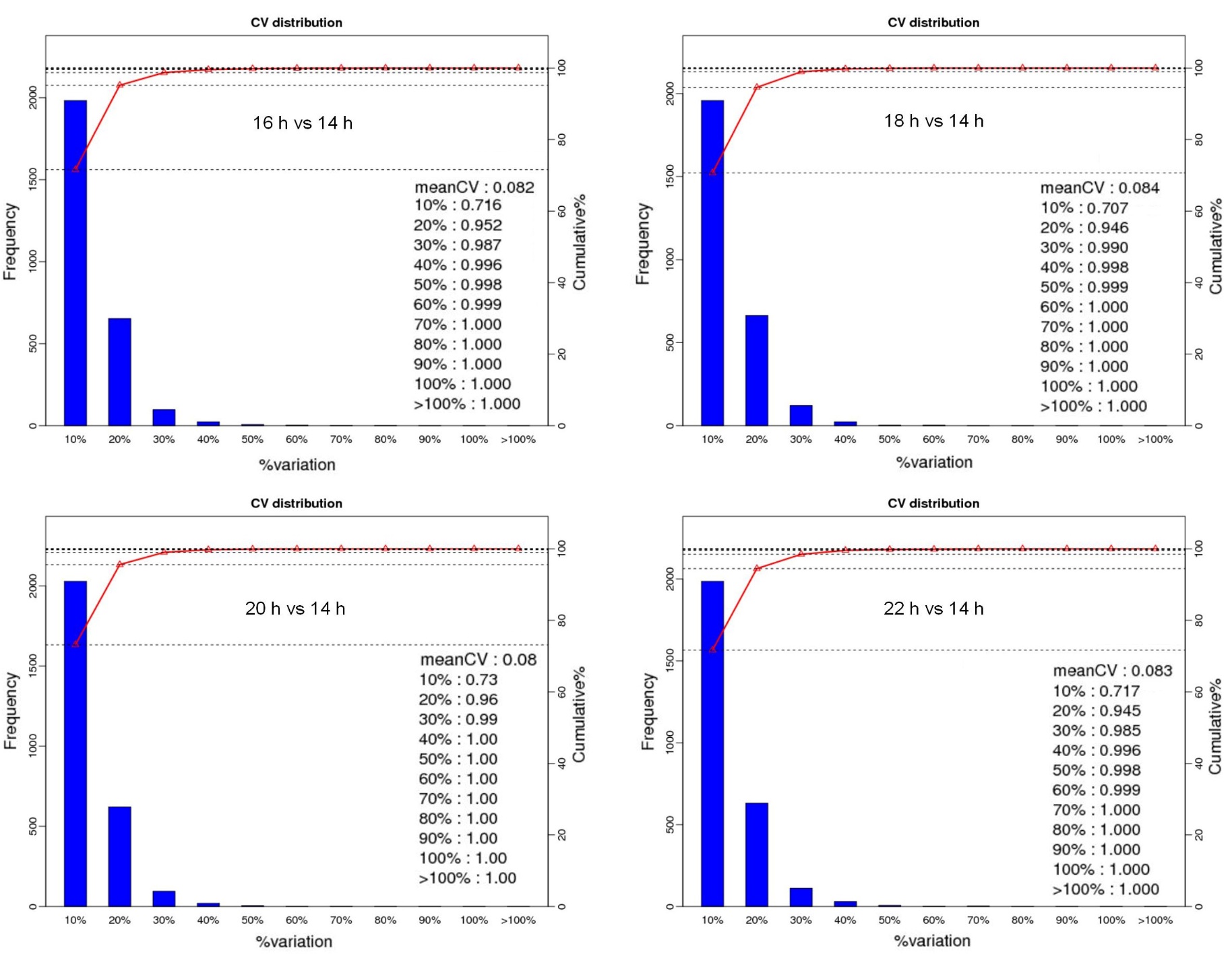


**Fig. S6** *CV* distribution in replicates. X-axis is the deviation between the protein ratio of the repetitive samples. Y-axis is the percentage that protein at a certain angle comprise quantified protein amount


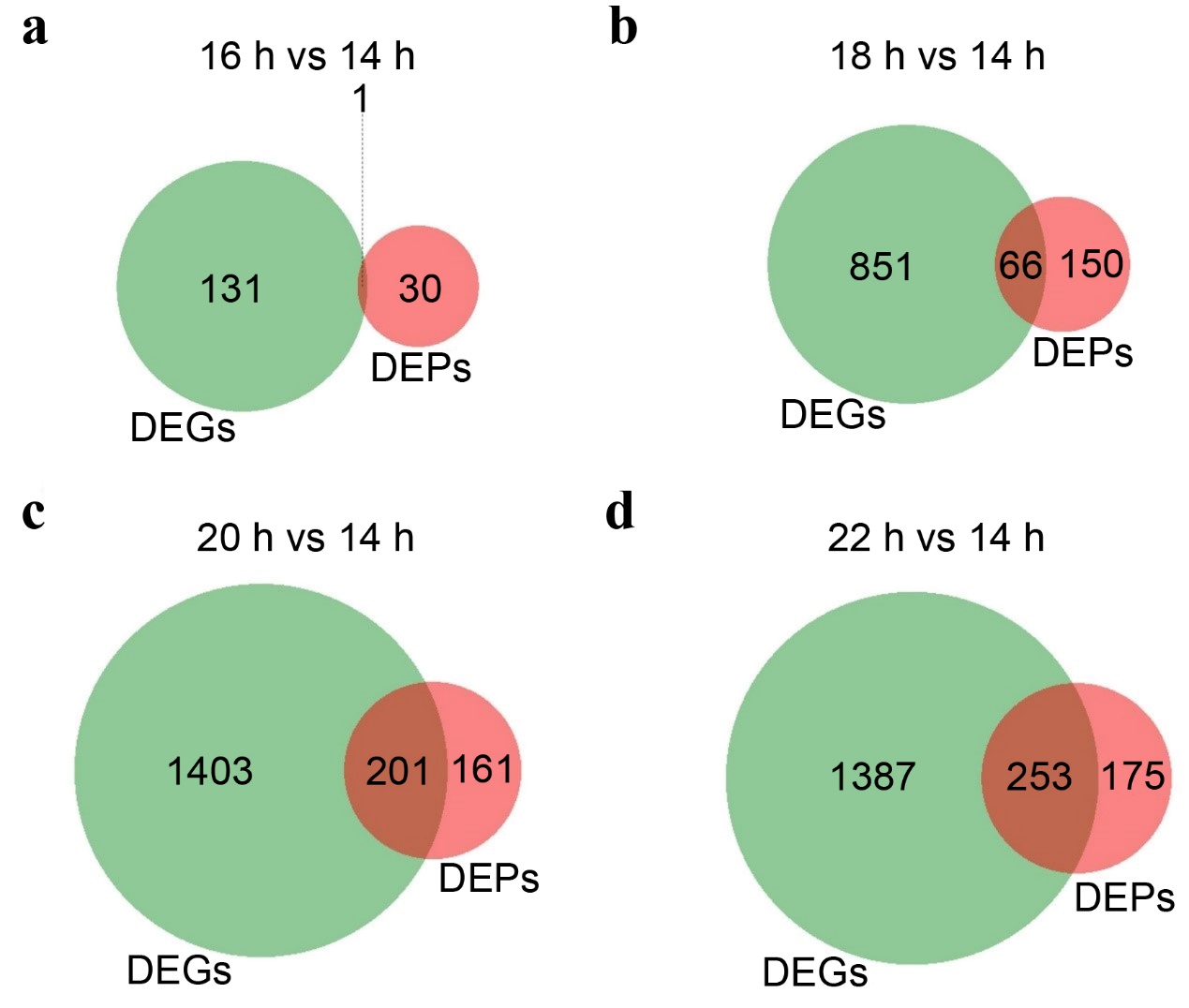


**Fig. S7** Venn diagrams showing overlaps between DEPs and DEGs

**
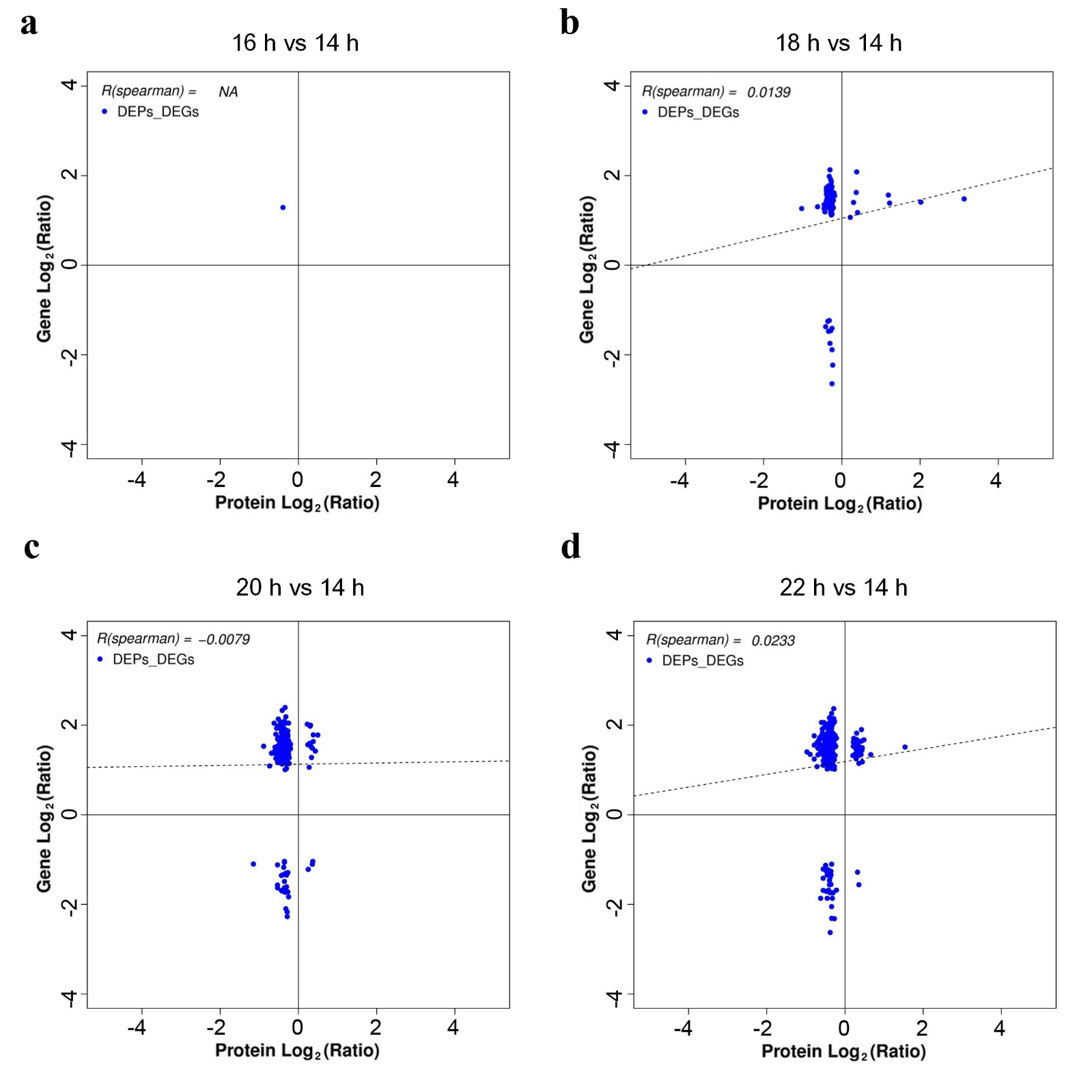
**

**Fig. S8** Spearman correlation analysis between proteome and transcriptome based on the data of DEPs and the correlated DEGs. The absolute value of an *R(Spearman)* between 0.5 and 1 indicates a strong correlation. The greater the absolute value of correlation coefficient, the stronger the correlation

**
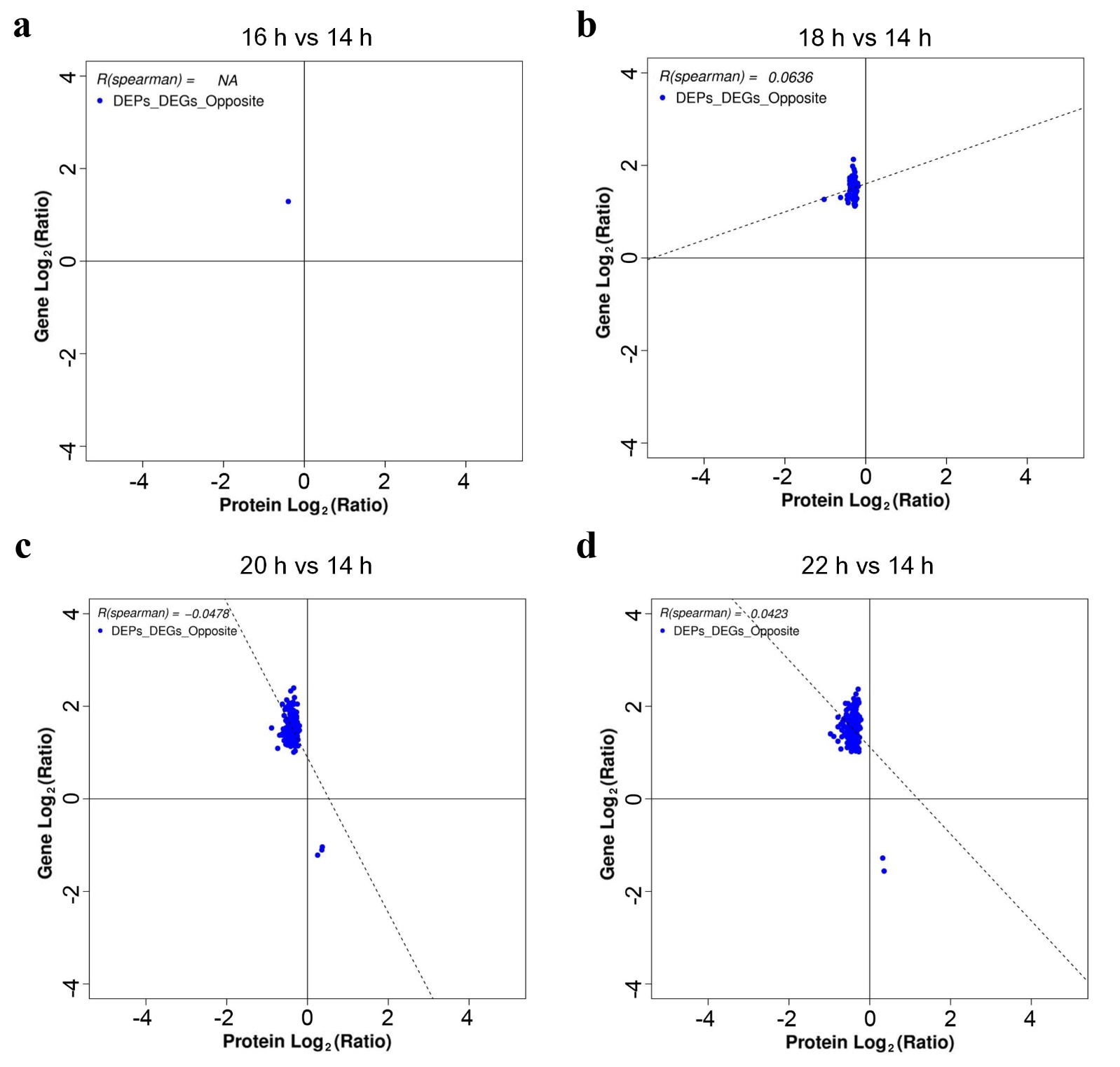
**

**Fig. S9** Spearman rank correlation analysis between proteome and transcriptome based on the data of DEPs with opposite expression trends to the correlated DEGs. The absolute value of an *R(Spearman)* between 0.5 and 1 indicates a strong correlation. The greater the absolute value of correlation coefficient, the stronger the correlation

**
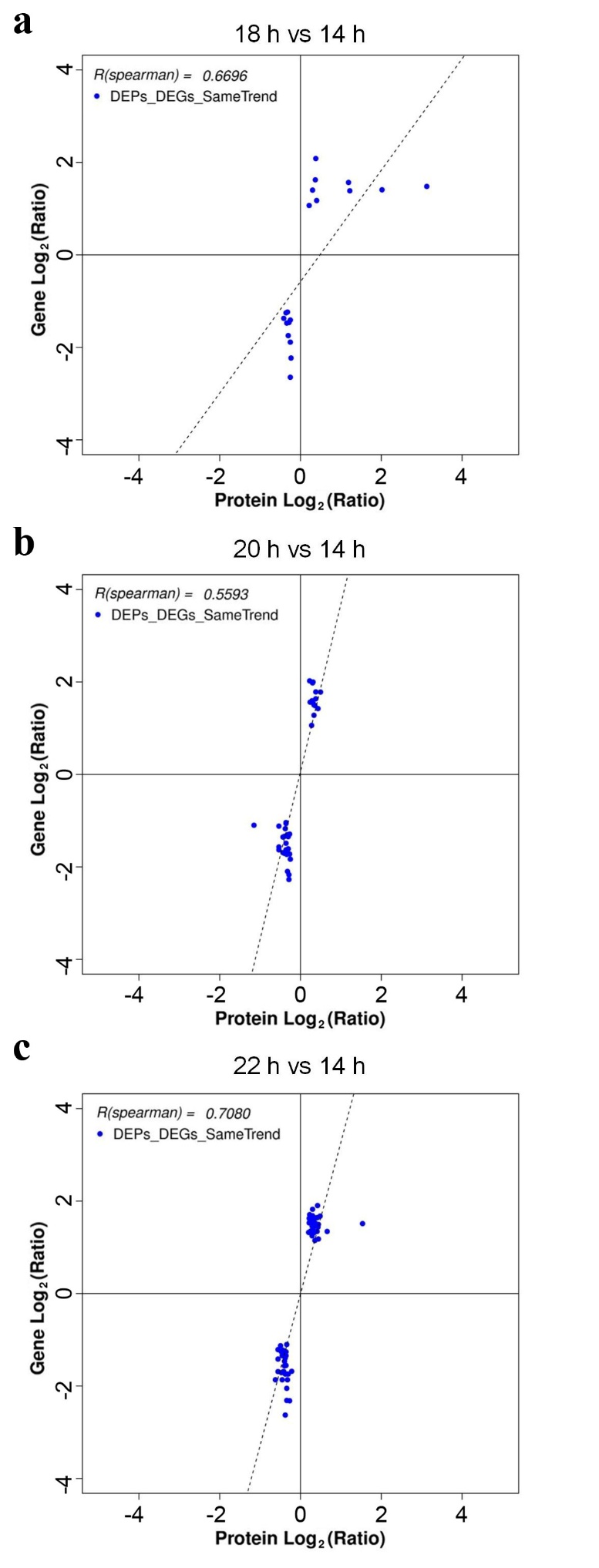
**

**Fig. S10** Spearman correlation analysis between proteome and transcriptome based on the data of DEPs with the same expression trends as the correlated DEGs. The absolute value of an *R(Spearman)* between 0.5 and 1 indicates a strong correlation. The greater the absolute value of correlation coefficient, the stronger the correlation. Given that no correlation was found between the DEPs and the DEGs with the same expression trends for 16 h vs 14 h, Spearman correlation test could not be conducted for this comparative group

**
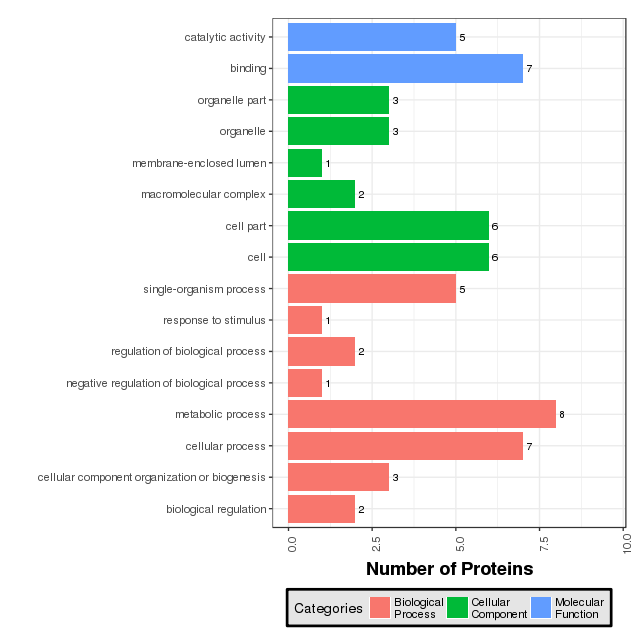
**

**Fig. S11** GO annotation of the DEPs with the same expression trends as DEGs for 18h vs 14s

**
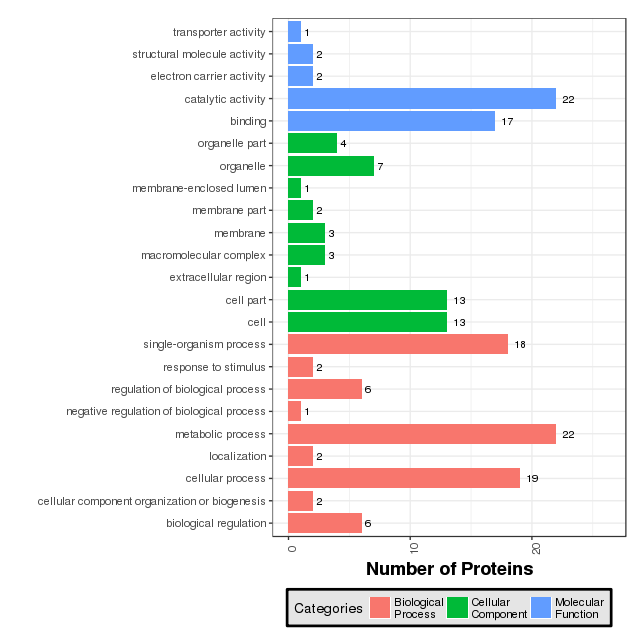
**

**Fig. S12** GO annotation of the DEPs with the same expression trends as DEGs for 20h vs 14s

**
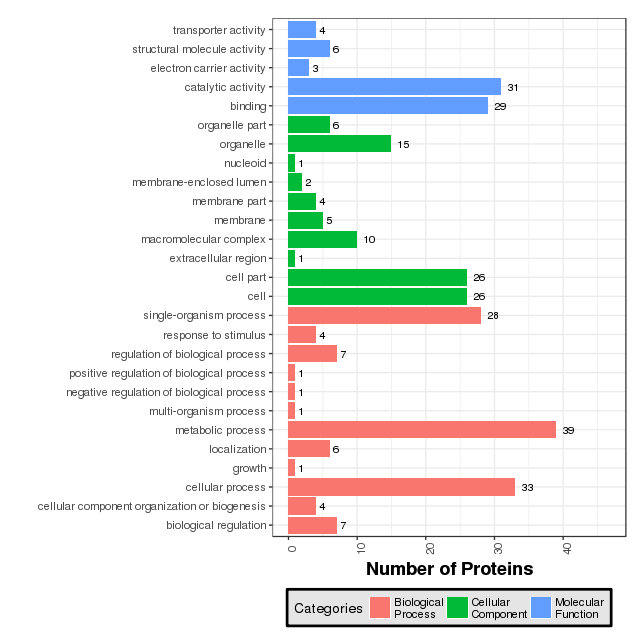
**

**Fig. S13** GO annotation of the DEPs with the same expression trends as DEGs for 22h vs 14s


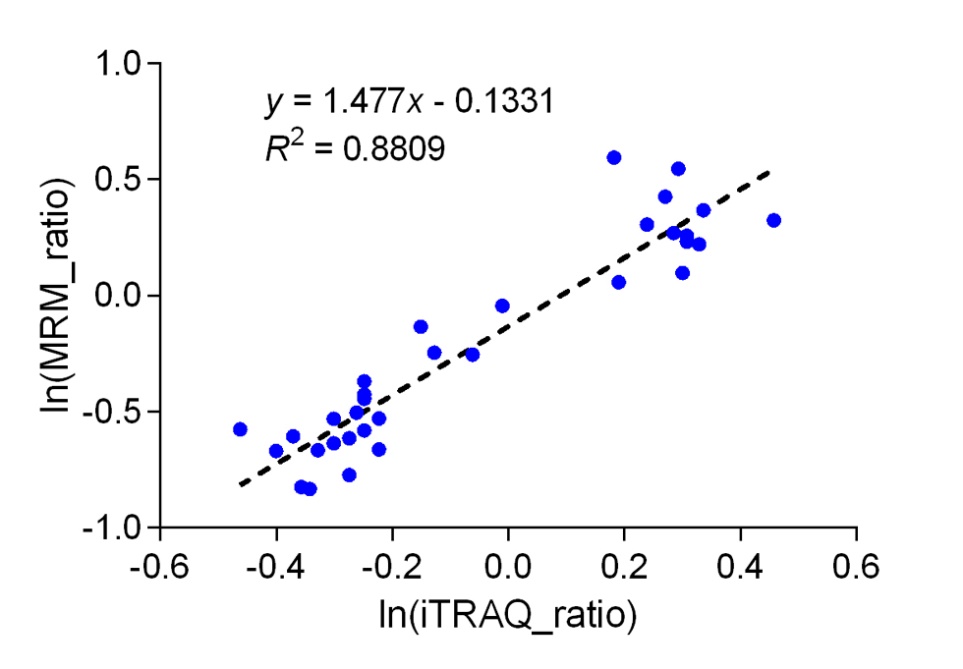


**Fig. S14** Scatter plot of iTRAQ quantified ln(protein ratio) and MRM quantified ln(protein ratio) for 22 h vs 14 h. ln(iTRAQ_ratio), iTRAQ quantified ln(protein ratio); ln(iTRAQ_ratio), MRM quantified ln(protein ratio)
